# Immortal time bias: a possible explanation for “Impact of acyclovir use on survival of patients with ventilator-associated pneumonia and high load herpes simplex virus replication”

**DOI:** 10.1186/s13054-020-03073-4

**Published:** 2020-06-17

**Authors:** Magnus Glindvad Ahlström, Lars Haukali Omland, Andreas Ronit, Niels Obel

**Affiliations:** 1 Department of Clinical Microbiology, Copenhagen University Hospital, Rigshospitalet, Copenhagen, Denmark; 2 Department of Infectious Diseases, Copenhagen University Hospital, Rigshospitalet, Copenhagen, Denmark; 3 Department of Infectious Diseases, Copenhagen University Hospital, Hvidovre Hospital, Hvidovre, Denmark

Dear editor,

We have with great interest read the paper “Impact of acyclovir use on survival of patients with ventilator-associated pneumonia and high load herpes simplex virus replication” published recently in *Critical Care* [1]. The study describes impact of acyclovir treatment on mortality in patients with ventilator-associated pneumonia who tested positive for herpes simplex virus in respiratory secretions. The authors conclude that treatment with acyclovir in patients with high HSV viral load decreases mortality in this population dramatically.

The authors address an important scientific question. However, we have concerns regarding the statistical analyses of survival. The authors seem to separate patients in acyclovir treated and not treated patients. This statistical strategy may lead to immortal time bias, which is a bias that results in substantial overestimation of the effect of medical treatment [2, 3]. Even for medical treatment without effect, that bias may lead to large estimates that are “statistically significant”. We identified potential scenarios in the current study in which immortal time bias could occur: (1) acyclovir treatment precedes HSV-positive test leading to immortal time included in the acyclovir treated group (Fig. 1a) and (2) HSV-positive test precedes acyclovir treatment leading to immortal time excluded from the untreated group (Fig. 1b), both of which would lead to an “artificial” survival advantage for the treated group.

**Fig. 1:**
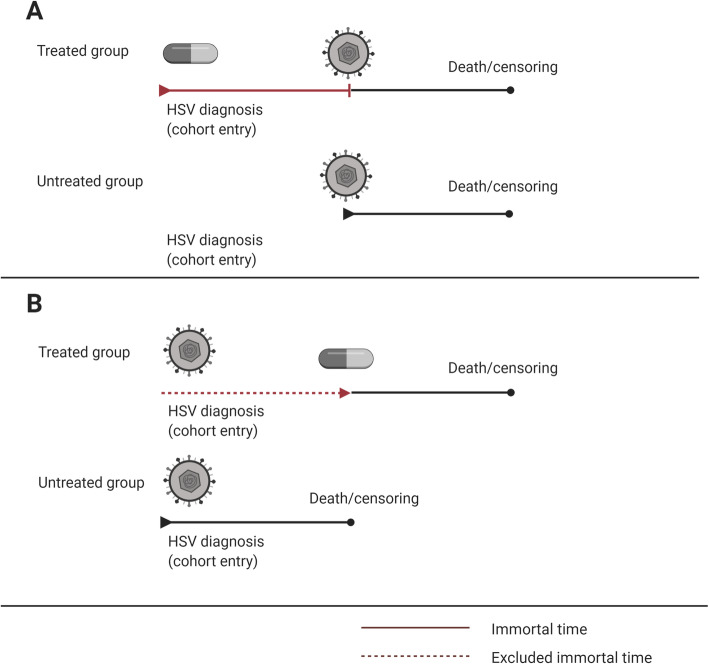
Potential immortal time bias in the current study. **a** Acyclovir treatment preceding HSV test, time is started at acyclovir treatment leading to an immortal time from start of treatment to HSV test. **b** HSV test preceding acyclovir treatment leading to excluded immortal time from the untreated group

We therefore suggest that the authors reanalyze their data, in which they start observation at the date of test for herpes simplex virus and include acyclovir treatment as a time updated exposure.

## Data Availability

Not applicable.
